# Low dietary calcium intake leads to a higher colorectal cancer burden in countries with low social development: findings from the Global Burden of Disease Study 2021

**DOI:** 10.3389/fnut.2025.1545085

**Published:** 2025-06-03

**Authors:** Junhong Wu, Ziyi Zhang, Jiong Chen, Shiya Yu, Donglin Liu, Junhui Jiang, Tingting Liu, Hu Zhao, Yu Wang

**Affiliations:** ^1^Department of General Surgery, Fuzong Clinical Medical College of Fujian Medical University, 900th Hospital of PLA Joint Logistic Support Force, Fuzhou, China; ^2^The First Clinic Center, 900th Hospital of PLA Joint Logistic Support Force, Fuzhou, China; ^3^Department of General Surgery, Fuzhou General Teaching Hospital, Fujian University of Traditional Chinese Medicine, 900th Hospital of PLA Joint Logistic Support Force, Fuzhou, China

**Keywords:** colorectal cancer, epidemiological burden, calcium intake, GBD 2021, disability-adjusted life years

## Abstract

**Background:**

Numerous studies have demonstrated that individuals with low calcium intake are at increased risk of developing colorectal cancer (CRC), and calcium intake exhibits significant global variation. However, a comprehensive analysis of the diet low in calcium-attributable colorectal cancer (DLCACRC) disease burden remains lacking.

**Objective:**

This study aimed to investigate the global distribution and temporal trends of DLCACRC from 1990 to 2021, providing evidence to support the development of evidence-based nutrition policies. Methods: Data on deaths, disability-adjusted life years (DALYs), mortality rates, and DALYs of DLCACRC between 1990 and 2021 were extracted from the GBD database. Age-standardized data were utilized to facilitate comparisons across regions and countries. Joinpoint regression analysis was conducted to assess temporal patterns in disease burden. Estimated annual percentage changes (EAPCs) were calculated to quantify the rate of change in relevant indicators. Pearson correlation analysis was performed to determine the relationship between the disease burden and the Social Development Index (SDI).

**Result:**

In 2021, the global age-standardized mortality rate (ASMR) of DLCACRC reached 1.06 (95% CI: 0.77–1.33), while the age-standardized disability-adjusted life year rate (ASDR) was 24.7 (95% CI: 18.17–31.02). These metrics demonstrated a downward trend, showing 31.3 and 33.3% reductions, respectively, compared to 1990. The most rapid reductions in ASMR and ASDR were occurred during 2004 and 2007, with annual percentage change (APC) of −2.12 (95% CI: −2.80–1.43) and −2.29 (95% CI: −2.92–1.65), respectively. Significant differences in disease burden were observed across countries and regions, with Southeast Asia reporting the highest ASMR and ASDR of DLCACRC. At the national level, Zambia recorded the highest ASMR and ASDR. Women experienced a higher disease burden than men, and the disease burden was positively correlated with age.

**Conclusion:**

From 1990 to 2021, the global disease burden of DLCACRC declined, although substantial regional disparities persist. Governments in these regions should adopt targeted strategies to enhance calcium intake among residents, thereby alleviating the disease burden. Particular attention should be given to women and older adults.

## 1 Introduction

Colorectal cancer (CRC) is the third most prevalent malignancy globally and the second leading cause of cancer-related mortality. In 2020, approximately 1.93 million new cancer cases were reported globally, with CRC accounting for around 10.0%, resulting in an estimated 0.94 million deaths, which represented 9.4% of all cancer-related deaths (1). Although the incidence of CRC varies significantly across global regions, both the number of newly diagnosed cases and the age-standardized incidence rate (ASIR) have shown a steady increase (2). A systematic analysis from the Global Burden of Disease Study 2017 evaluated global trends in CRC across 195 countries and territories from 1990 to 2017. The results indicate that highly developed countries achieved stabilization or even reductions in CRC incidence and mortality (3). However, many low- and middle-income countries were experiencing a rapid increase (4).

Colorectal carcinogenesis is multifactorial, involving genetic predisposition, advancing age, environmental factors, lifestyle determinants (particularly dietary patterns and physical activity), and metabolic risks (5). However, the exact etiology of CRC remains unclear, and these risk factors may introduce potential confounding effects, leading to misleading associations in observational studies. Among the many established risk factors for CRC, diet played a crucial role, and optimizing dietary habits is considered an effective and modifiable strategy for reducing the CRC burden (6). The 2021 Global Burden of Disease (GBD) Study identified the top five dietary factors associated with CRC risk: insufficient whole grain intake, inadequate dairy intake, excessive red meat consumption, insufficient calcium intake, and excessive unprocessed meat consumption. According to the 2019 GBD study, dietary factors were recognized as key determinants influencing the prognosis of CRC, with dietary components affecting CRC through multiple mechanisms (7–9).

Calcium is one of the essential minerals required by the human body, playing a critical role in supporting nerve conduction, muscle function, blood coagulation, and other physiological functions (10–13). A study by the American Cancer Society on cancer prevention showed that individuals who consumed the most calcium through diet and supplements had a lower risk of CRC compared to those with the lowest calcium intake. Individuals who consumed more than 700 milligrams of calcium per day had a 35 to 45 percent lower risk of distal colon cancer compared to those consuming less than 500 milligrams per day (14, 15). The global average dietary calcium intake ranged from 175 to 1,233 mg/day, with considerable variation across regions. Insufficient calcium intake predominantly occurred in countries with lower levels of socioeconomic development (16, 17).

Differences in dietary calcium intake affect individual nutritional profiles and may influence the progression of colorectal cancer to varying degrees. Diet low in calcium-attributable colorectal cancer (DLCACRC) is defined as colorectal malignancy occurring in individuals whose daily dietary calcium intake falls below the thresholds established by international guidelines. Investigating the disease burden of DLCACRC across different regions may support the development of national cancer prevention strategies and promote healthier dietary practices. However, there remains a lack of systematic and relevant epidemiological studies. The GBD database was currently the most comprehensive and reliable source of disease burden data worldwide (18). This study quantified the global, regional, and national disease burden of DLCACRC by retrieving relevant data from the GBD 2021 and analyzing the trends over time, as well as gender, age, and regional differences. The findings are summarized below.

## 2 Materials and methods

### 2.1 Data source

The data used in this study were obtained from GBD 2021 via the Global Health Data Exchange (GHDx) query tool (18). The GBD database integrates information from diverse data sources across 204 countries worldwide, including vital registration, verbal autopsy, population census, household surveys, disease-specific registries, and health service data, providing detailed data on 371 diseases. To correct biases in the source data, the Bayesian mixed-effects meta-regression model DisMod-MR 2.1 was applied. This model, developed by the Institute for Health Metrics and Evaluation (IHME) in Seattle, Washington, USA, serves as a robust framework for estimating the burden of diseases, injuries, and risk factors (19, 20). This study collected and analyzed data on DLCACRC across 7 super-regions, 21 regions, 204 countries and territories (including 21 countries with sub-national levels), and 811 subnational units spanning the years 1990 to 2021. The data included the number of deaths, disability-adjusted life years (DALYs), and age-standardized rate (ASR). All data were systematically stratified by sex, age, and geographic region. According to the Socio-demographic Index (SDI), countries and regions were categorized into five levels: high, high-middle, middle, low-middle, and low. Simultaneously, based on geographical location, the 204 countries and regions were further categorized into 21 regions. Additionally, individuals were categorized into 12 age groups at 5-year intervals, starting from age 25.

### 2.2 Definitions

Low dietary calcium intake was defined as a daily calcium intake of less than 1.06 to 1.1 grams from all food sources, including calcium-rich foods such as cheese, milk, and yogurt (21). DALYs were defined as the total number of healthy years of life lost due to morbidity and mortality, calculated as the sum of years of life lost due to premature mortality (YLL) and years of life lost due to disability (YLD). YLLs were calculated by multiplying the number of cause-age-sex-location-year-specific deaths by the difference between the standard life expectancy and the age at death. YLDs were calculated by multiplying the cause-age-sex-location-year-specific prevalence of sequelae by their corresponding disability weights and the duration of the condition. Mortality rates were defined as the proportion of deaths from a specific disease or injury per 100,000 population during a specified time period. ASR was defined as the ratio calculated based on the standard age composition of the population. SDI is a composite indicator used to comprehensively assess a country’s level of development, based on three dimensions: national education level, total fertility rate, and per capita income. Specifically, the SDI is calculated as the geometric mean of three components: lag-distributed income per capita, average years of education, and the total fertility rate among women younger than 25 years. This index reflects the overall socioeconomic development status of a country or region (22).

### 2.3 Statistical analysis

When comparing countries and regions, the age-standardized mortality rate (ASMR) and the age-standardized disability-adjusted life rate (ASDR) are employed to eliminate bias caused by differences in population age structures. The formula for calculating the ASR was as follows:

$\mathrm{ASR}=\frac{\sum_{i=1}^{A}a_{i}w_{i}}{\sum_{i}^{A}w_{i}}\times 100,000.$

Where *a_i_* represented the age-specific rate for the *i*-th age group, *w* denotes the number of people in each *i*-th age group in the selected standard population, and *A* referred to the number of age groups. The units were expressed per 100,000 people based on the global standard population (23).

The Joinpoint Regression Program (version 5.0) was employed to analyze global change trends. This model subdivides the long-term trend line into multiple intervals and fits and optimizes the trend for each interval. Using this model, the annual percentage change (APC) and its 95% confidence interval (95% CI) for each interval can be calculated, thereby enabling a comprehensive evaluation of both the overall change trend across the entire time range and the characteristic changes in different intervals (24).

The estimated annual percentage change (EAPC) was employed to assess the rate of change for each indicator across 204 countries and regions worldwide from 1990 to 2019. The calculation formula is:


$$\text{EAPC}=(e^{\beta }-1)$$

In this equation, *β* represented the regression coefficient of the log-transformed ASR in relation to the year, as obtained through the linear regression model. Specifically, assuming a linear relationship between the natural logarithm of the ASR and the year, the equation is expressed as: ln(ASRt) = *α* + *β*t + *σ*, where α is the intercept, *β* was the slope (i.e., the regression coefficient), and σ was the error term. The 95% confidence interval (CI) is derived from the linear regression model. EAPC reflected the average annual percentage change of a health indicator over a specific time period. A positive EAPC indicated an increasing trend in the indicator over time, while a negative EAPC suggested a decreasing trend.

Given that ASMR, ASDR, and SDI were continuous variables, Pearson correlation analysis was used to assess the relationship between ASR and SDI across 204 countries and 21 regions.

Microsoft Excel and R 4.4.2 software were used to clean and organize the data obtained from the GBD database. Statistical analysis and drawing were performed using R 4.4.2 software after installing ggplot2, dplyr, reshape2 and other packages.

## 3 Results

### 3.1 DLCACRC burden and its trends

In 2021, the global number of deaths and DALYs of DLCACRC were 89,089 (95% CI: 65,019–112,298) and 2,128,939 (95% CI: 1,565,530–2,672,450), respectively, representing 8.5 and 8.7% of the total CRC-related deaths and DALYs. From 1990 to 2021, the global number of deaths of DLCACRC increased by 55.3%, rising from 57,363 (95% CI: 42,915–71,257) to 89,089 (95% CI: 65,019–112,298). Concurrently, DALYs also increased by 40.7%, from 1,512,762 (95% CI: 1,132,026–1,881,667) to 2,128,939 (95% CI: 1,565,530–2,672,450) (Table 1).

**Table 1:** Global burden of DLCACRC in 1990 and 2021 for both sexes and all locations, with EAPC.

Characteristics	1990				2021				EAPC(1990–2019)	
	Deaths cases	ASMR per 100,000	DALYs	ASDR per 100,000	Deaths cases	ASMR per 100,000	DALYs	ASDR per 100,000	ASMR per 100,000	ASDR per 100,000
	No. (95%CI)	No. (95%CI)	No. (95%CI)	No. (95%CI)	No. (95%CI)	No. (95%CI)	No. (95%CI)	No. (95%CI)	No. (95%CI)	No. (95%CI)
Global	57,363 (42,915, 71,257)	1.54 (1.15, 1.92)	1,512,762 (1,132,026, 1,881,667)	37.04 (27.74, 46.14)	89,089 (65,019, 112,298)	1.06 (0.77,1.33)	2,128,939 (1,565,530, 2,672,450)	24.7 (18.17, 31.02)	−1.33 (−1.37, −1.29)	−1.45 (−1.5, −1.4)
Male	23,407 (17,131, 30,625)	1.37 (1, 1.8)	657,404 (478,504, 854,471)	33.79 (24.47, 44.07)	36,968 (26,842, 49,073)	0.96 (0.69, 1.27)	939,669 (680,055, 1,241,878)	22.95 (16.61, 30.33)	−1.26 (−1.32, −1.2)	−1.36 (−1.41, −1.31)
Female	33,956 (24,765, 42,701)	1.65 (1.2, 2.08)	855,358 (617,926, 1,075,506)	39.69 (28.74, 49.84)	52,121 (37,283, 66,521)	1.13 (0.81, 1.44)	1,189,269 (865,714, 1,502,083)	26.13 (19.01, 32.99)	−1.37 (−1.42, −1.32)	−1.51 (−1.57, −1.45)
Southeast Asia	8,816 (6,634, 10,796)	3.64 (2.77, 4.44)	561,490 (402,571, 735,058)	60.93 (43.56, 79.33)	20,991 (15,708, 26,106)	3.41 (2.56, 4.23)	536,168 (379,605, 725,187)	25.05 (17.63, 33.9)	−0.32 (−0.4, −0.24)	−3.01 (−3.11, −2.92)
East Asia	19,435 (13,891, 25,176)	2.52 (1.79, 3.24)	256,099 (191,960, 315,041)	91.45 (68.83, 112.07)	22,174 (15,483, 29,949)	1.07 (0.75, 1.45)	560,315 (418,460, 695,240)	81.73 (61.48, 101.51)	−2.9 (−2.99, −2.8)	−0.49 (−0.57, −0.41)
Oceania	34 (24,45)	1.36 (1.01, 1.76)	1,045 (750, 1,384)	32.29 (22.88, 42.42)	80 (57, 100)	1.18 (0.85, 1.47)	2,413 (1738, 3,087)	28.53 (20.63, 36.04)	−0.41 (−0.45, −0.37)	−0.35 (−0.38, −0.32)
Central Asia	304 (221, 387)	0.67 (0.48, 0.85)	8,346 (6,063, 10,715)	17.09 (12.4, 21.91)	300 (209, 398)	0.4 (0.28, 0.52)	8,006 (5,482, 10,699)	9.42 (6.49, 12.56)	−1.94 (−2.13, −1.75)	−2.31 (−2.53, −2.09)
Central Europe	1,530 (1,074, 2016)	1.07 (0.75, 1.41)	33,949 (23,870, 44,795)	22.97 (16.15, 30.26)	2067 (1,434, 2,743)	0.89 (0.61, 1.18)	39,956 (27,821, 53,315)	18.24 (12.66, 24.39)	−0.95 (−1.21, −0.69)	−1.05 (−1.31, −0.79)
Eastern Europe	2,929 (2,109, 3,786)	1.07 (0.77, 1.38)	70,756 (50,948, 91,823)	25.33 (18.22, 32.83)	3,086 (2,107, 4,050)	0.86 (0.59, 1.13)	64,953 (44,555, 85,534)	18.68 (12.79, 24.61)	−1.38 (−1.67, −1.09)	−1.75 (−2.07, −1.43)
High-income Asia Pacific	2,643 (1,934, 3,365)	1.39 (1.02, 1.77)	61,786 (45,437, 78,698)	30.8 (22.66, 39.2)	5,967 (4,201, 7,921)	1.04 (0.74, 1.36)	98,291 (70,305, 129,309)	21.6 (15.46, 28.19)	−0.8 (−0.88, −0.73)	−1.03 (−1.1, −0.95)
Australasia	247 (171, 327)	1.08 (0.75, 1.42)	5,235 (3,594, 6,927)	22.65 (15.57, 29.95)	361 (243, 494)	0.62 (0.42, 0.85)	6,665 (4,495, 9,184)	12.75 (8.58, 17.42)	−1.99 (−2.15, −1.83)	−2.09 (−2.25, −1.93)
Western Europe	5,562 (3,881, 7,425)	0.92 (0.65, 1.23)	104,399 (73,127, 139,614)	18.11 (12.7, 24.23)	5,299 (3,558, 7,159)	0.48 (0.33, 0.65)	89,755 (61,109, 120,994)	9.61 (6.56, 12.96)	−2.07 (−2.14, −1.99)	−2.01 (−2.09, −1.94)
Southern Latin America	646 (473, 824)	1.48 (1.08, 1.88)	13,972 (10,156, 17,951)	30.55 (22.21, 39.19)	1,013 (724, 1,340)	1.13 (0.81, 1.5)	20,622 (14,724, 27,361)	23.89 (17.1, 31.67)	−0.46 (−0.63, −0.3)	−0.37 (−0.52, −0.22)
High-income North America	3,040 (2,102, 4,076)	0.84 (0.58, 1.12)	58,581 (40,963, 78,633)	16.73 (11.68, 22.4)	2,972 (2039, 4,033)	0.43(0.3, 0.59)	58,618 (40,740, 79,326)	9.43 (6.62, 12.7)	−2.01 (−2.11, −1.91)	−1.67 (−1.79, −1.54)
Caribbean	404 (298, 508)	1.62 (1.21, 2.03)	9,783 (7,226, 12,441)	37.18 (27.42, 47.2)	787 (575, 1,022)	1.46 (1.07, 1.9)	18,308 (13,185, 24,369)	34.24 (24.61, 45.57)	−0.45 (−0.53, −0.36)	−0.36 (−0.44, −0.29)
Andean Latin America	300 (219, 388)	1.57 (1.16, 2.04)	7,287 (5,299, 9,422)	34.56 (25.21, 44.85)	696 (478, 936)	1.21 (0.83, 1.63)	15,728 (10,586, 21,443)	26.36 (17.75, 35.89)	−0.99 (−1.09, −0.88)	−1.06 (−1.17, −0.96)
Central Latin America	629 (475, 775)	0.83 (0.63, 1.03)	15,637 (11,833, 19,220)	17.99 (13.58, 22.13)	1923 (1,396, 2,465)	0.79 (0.57, 1.01)	46,981 (34,242, 60,391)	18.49 (13.45, 23.76)	−0.23 (−0.36, −0.1)	0.01 (−0.13, 0.15)
Tropical Latin America	745 (556, 933)	0.91 (0.67, 1.14)	19,081 (14,358, 23,859)	20.18 (15.13, 25.21)	1791 (1,276,2, 335)	0.71 (0.51, 0.93)	43,201 (30,830, 56,259)	16.66 (11.89, 21.7)	−0.98 (−1.09, −0.86)	−0.86 (−0.98, −0.74)
North Africa and Middle East	1,496 (1,099, 1907)	0.98 (0.73, 1.24)	42,058 (30,089, 53,647)	23.3 (16.91, 29.73)	3,086 (2,209, 3,999)	0.75 (0.53, 0.96)	82,416 (58,652, 108,454)	17.06 (12.19, 22.18)	−0.77 (−0.84, −0.71)	−0.97 (−1.02, −0.92)
South Asia	4,233 (3,169, 5,448)	0.79 (0.59, 1.02)	123,780 (92,061, 158,662)	19.66 (14.67, 25.23)	8,376 (6,120, 10,592)	0.61 (0.45, 0.77)	220,553 (161,659, 280,598)	14.37 (10.57, 18.28)	−0.88 (−0.94, −0.82)	−1.06 (−1.11, −1.01)
Central Sub-Saharan Africa	423 (304, 554)	2.18 (1.56, 2.81)	12,169 (8,770, 16,048)	51.15 (36.79, 66.95)	1,094 (726, 1,576)	2.29 (1.52, 3.39)	31,598 (20,564, 45,047)	52.97 (35.14, 76.47)	0.1 (0.04, 0.15)	0.04 (−0.01, 0.09)
Eastern Sub-Saharan Africa	2,219 (1,563, 2,810)	3.21 (2.28, 4.05)	62,679 (43,301, 79,618)	78.22 (54.98, 99.09)	3,627 (2,665, 4,547)	2.56 (1.91, 3.14)	95,984 (70,422, 123,976)	54.71 (40.17, 68.74)	−1.03 (−1.15, −0.92)	−1.52 (−1.66, −1.39)
Southern Sub-Saharan Africa	540 (411, 717)	2.19 (1.64, 2.91)	14,175 (10,755, 18,430)	49.53 (37.61, 65.11)	1,383 (1,054, 1703)	2.62 (1.97, 3.22)	36,721 (28,046, 45,674)	60.37 (46.28, 74.71)	0.57 (0.27, 0.87)	0.72 (0.4, 1.04)
Western Sub-Saharan Africa	1,186 (889, 1,488)	1.52 (1.16, 1.92)	30,455 (22,721, 38,391)	33.94 (25.36, 42.62)	2014 (1,447, 2,519)	1.22 (0.87, 1.49)	51,685 (36,709, 66,205)	25.6 (18.34, 32.19)	−0.77 (−0.82, −0.71)	−0.98 (−1.05, −0.91)
High-middle SDI	14,059 (10,251, 17,941)	1.5 (1.09, 1.91)	361,776 (263,117, 464,840)	35.97 (26.17, 46.12)	18,050 (12,759, 23,869)	0.92 (0.65, 1.22)	403,351 (287,676, 533,432)	20.79 (14.83, 27.52)	−1.74 (−1.83, −1.65)	−1.98 (−2.07, −1.89)
Low-middle SDI	7,334 (5,375, 9,339)	1.28 (0.96, 1.63)	235,254 (167,372, 309,405)	31.93 (23.29, 40.75)	14,888 (11,013, 18,210)	1.1 (0.82, 1.34)	276,182 (191,267, 367,830)	26.36 (19.35, 32.36)	−0.57 (−0.65, −0.5)	−1.48 (−1.52, −1.45)
High SDI	11,445 (8,119, 15,110)	1.03 (0.73, 1.36)	211,529 (154,145, 268,942)	21.54 (15.34, 28.32)	15,351 (10,701, 20,424)	0.66 (0.45, 0.87)	397,069 (291,742, 489,556)	13.44 (9.33, 17.88)	−1.45 (−1.48, −1.41)	−0.72 (−0.8, −0.65)
Low SDI	4,074 (2,951, 5,212)	1.96 (1.45, 2.47)	115,089 (82,738, 146,985)	47.66 (34.46, 60.97)	7,039 (5,238, 8,698)	1.59 (1.18, 1.97)	189,763 (140,383, 235,803)	35.54 (26.48, 43.95)	−0.87 (−0.97, −0.78)	−1.2 (−1.3, −1.1)
Middle SDI	20,392 (15,148, 25,325)	2.14 (1.59, 2.66)	587,646 (434,963, 736,458)	52.46 (38.97, 65.36)	33,662 (25,076, 42,327)	1.31 (0.97, 1.65)	860,241 (640,404, 1,080,803)	31.39 (23.35, 39.44)	−1.72 (−1.78, −1.66)	−1.8 (−1.86, −1.74)

**Figure 1:**
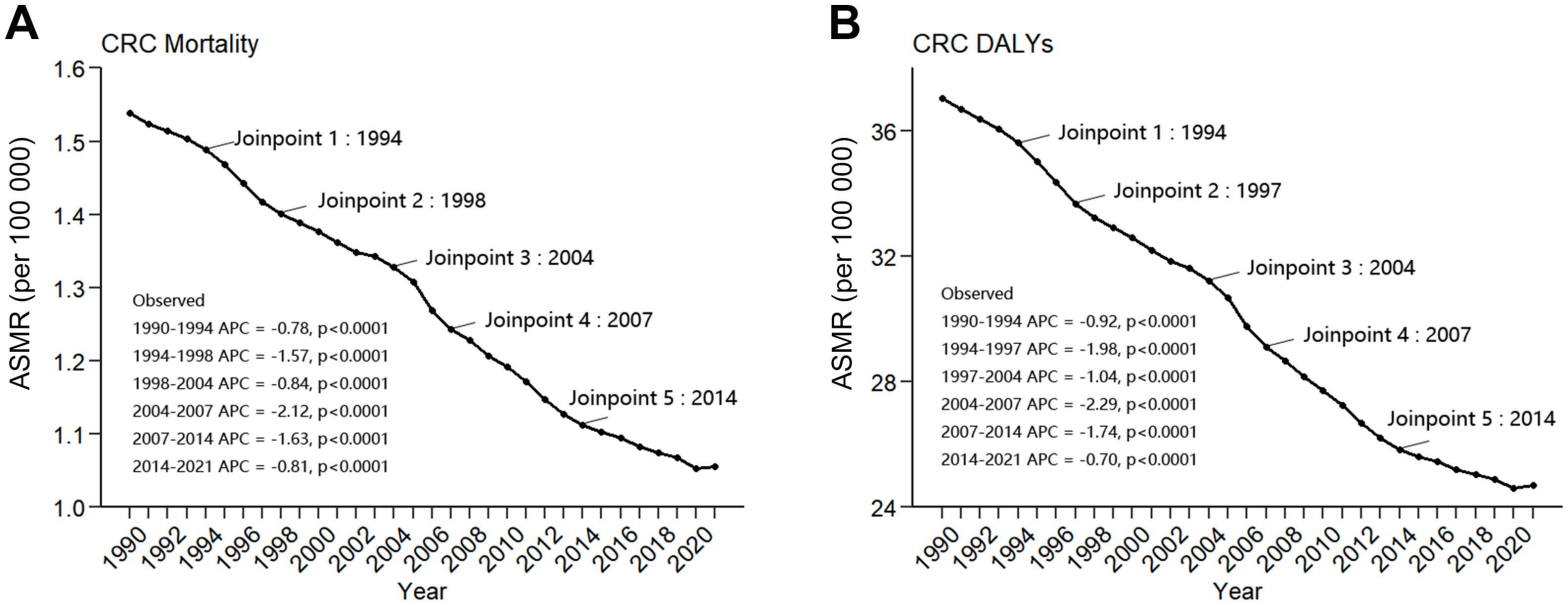
Joinpoint regression analysis of ASMR **(A)** and ASDR **(B)** of DLCACRC from 1990 to 2021.

Contrary to the trends in deaths and DALYs, the global ASMR and ASDR for DLCACRC exhibited a downward trend from 1990 to 2021. Specifically, the ASMR decreased by 31.3%, while the ASDR declined by 33.3%. Both measures declined at similar rates, with an EAPCs of −1.33 (95% CI = −1.37–−1.29) for ASMR and −1.45 (95% CI = −1.5–−1.4) for ASDR, respectively. In 2021, the global ASMR and ASDR for DLCACRC were 1.06 (95% CI = 0.77–1.33) and 24.7 (95% CI = 18.17–31.02), respectively (Table 1). The most pronounced decline in ASMR was observed between 2004 and 2007. During this period, the APC was −2.12 (95% CI = −2.80–−1.43). The most rapid decline in ASDR also occurred between 2004 and 2007, the APC was −2.29 (95% CI = −2.92–−1.65) (Figure 1).

### 3.2 Geographical differences of DLCACRC

From a geographical perspective, Southeast Asia had the highest ASMR for DLCACRC in 2021, at 3.41 (95% CI = 2.56–4.23), and also recorded the highest ASDR, at 25.05 (95% CI = 17.63–33.9), both approximately one-third higher than those observed in the second-highest region, sub-Saharan Africa. In the same year, Central Asia exhibited the lowest ASMR for DLCACRC, at 0.4 (95% CI = 0.28–0.52), and its ASDR was also the lowest, at 9.42 (95% CI = 6.49–12.56). Notably, the numerical differences in ASMR and ASDR among the three regions with the lowest rates were not substantial (Table 1 and Figure 1).

**Figure 2:**
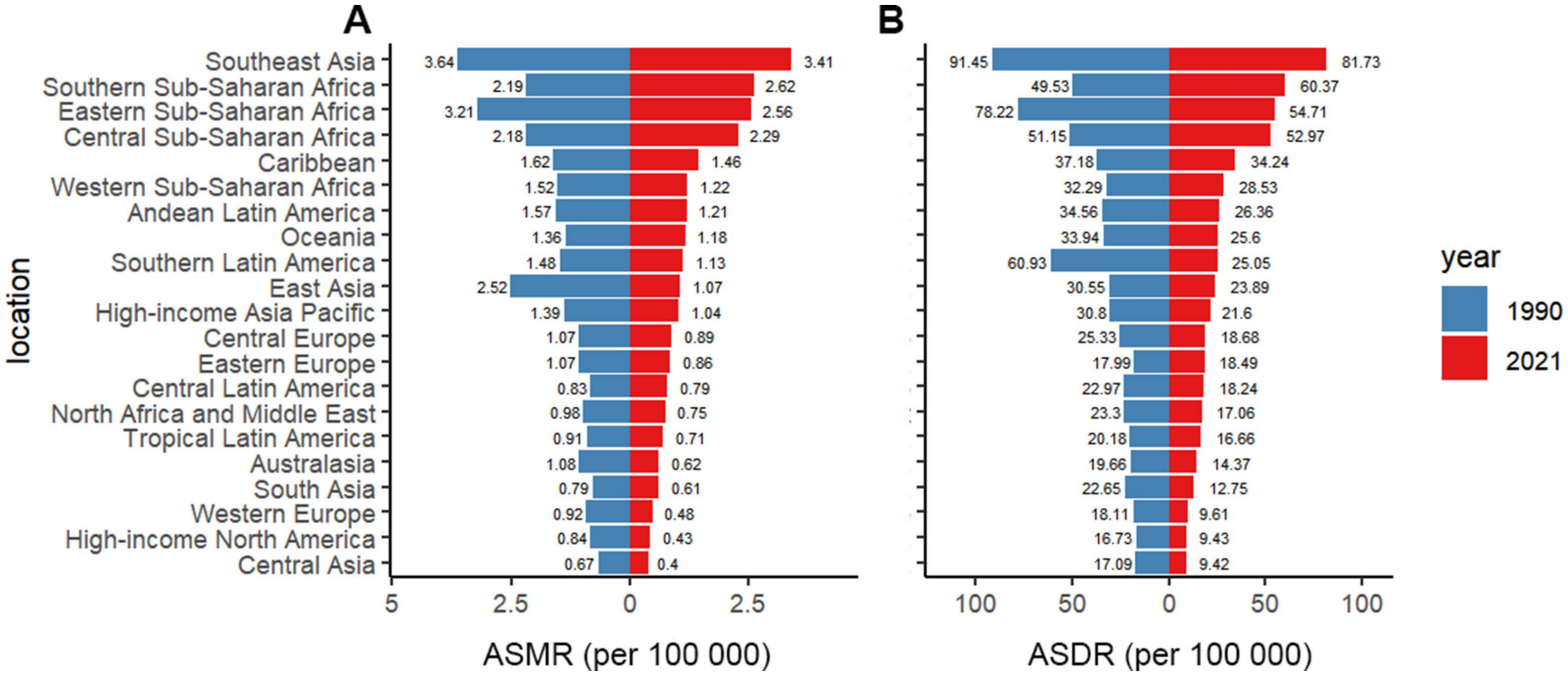
The ASMR **(A)** and ASDR **(B)** of DLCACRC in 21 regions in 1990 and 2021.

Since 1990, Southeast Asia has ranked first in both ASMR and ASDR, with values of 3.64 (95% CI = 2.77–4.44) and 60.93 (95% CI = 43.56–79.33), respectively. From 1990 to 2021, although both ASMR and ASDR in Southeast Asia declined, the rate of decrease was relatively slow, with the EAPCs for ASMR and ASDR at −0.32 (95% CI = −0.40–−0.24) and −0.49 (95% CI = −0.57–−0.41), respectively. Over the past three decades, both ASDR and ASMR have exhibited a declining trend in most regions. Among these regions, East Asia exhibited the most rapid decline, with EAPCs for ASMR and ASDR of −2.9 (95% CI = −2.99–-2.8) and −3.01 (95% CI = −3.11–−2.92), respectively. In contrast, ASMR and ASDR in Central and sub-Saharan Africa have shown an upward trend, particularly in sub-Saharan Africa, where the EAPCs for ASMR and ASDR are 0.57 (95% CI = 0.27–0.87) and 0.72 (95% CI = 0.4–1.04), respectively (Figure 2 and Table 1).

### 3.3 Country-level burden of DLCACRC

**Figure 3:**
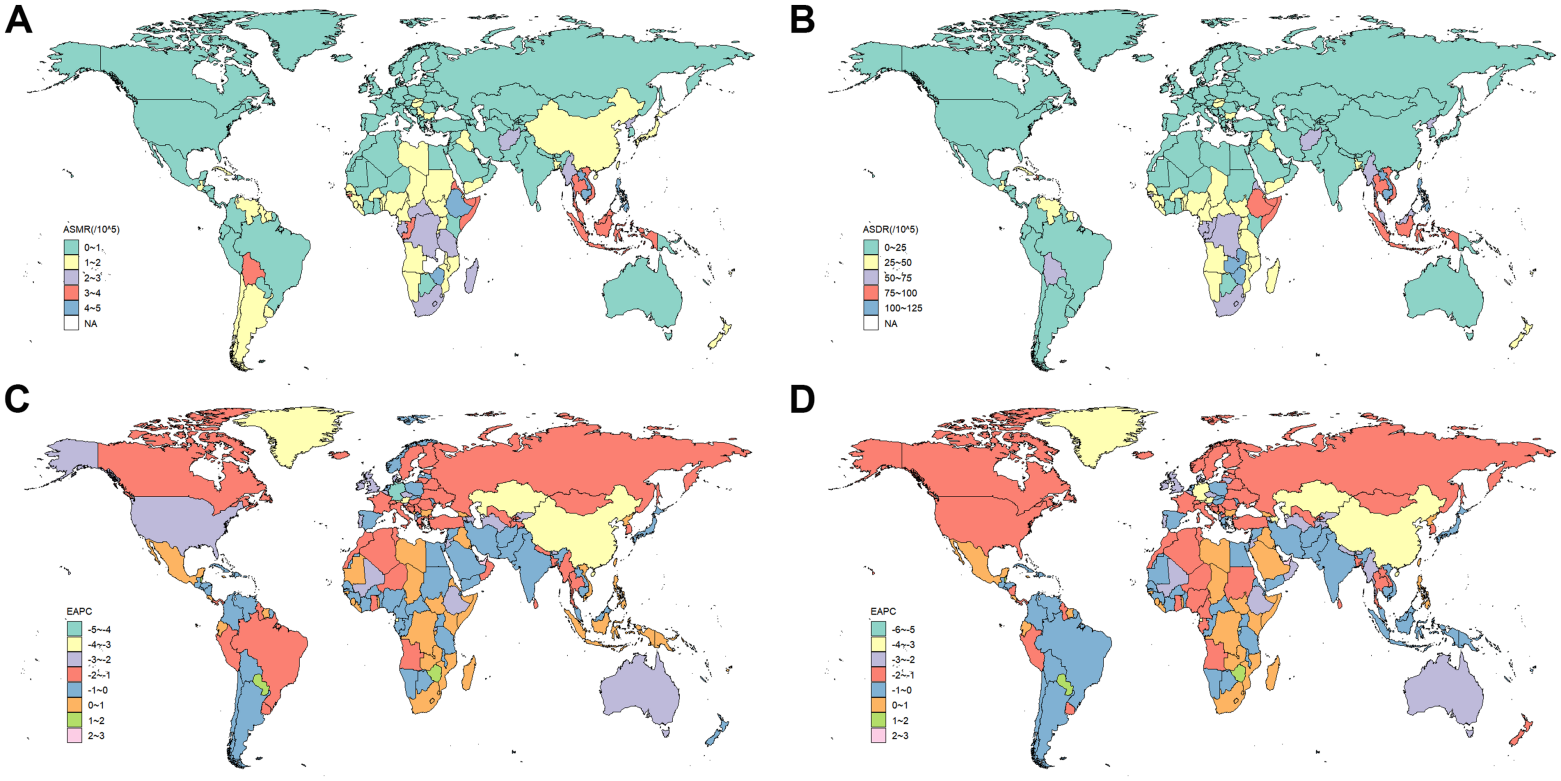
The ASMR and ASDR of DLCACRC and its EAPC value in 204 countries/territories in 2021. **(A)** The ASMR of DLCACRC in 204 countries/territories in 2021. **(B)** The ASDR of DLCACRC in 204 countries/territories in 2021. **(C)** The EAPC value of ASMR of DLCACRC in 204 countries/territories in 2021. **(D)** The EAPC value of ASMR of DLCACRC in 204 countries/territories in 2021.

In 2021, China ranked first globally in both the number of deaths and DALYs of DLCACRC, with 20,719 deaths (95% CI: 14,553–28,272) and 500,468 DALYs (95% CI: 356,219–682,873). Indonesia and India ranked second and third, respectively (Supplementary Tables 1, 2). In the ASMR and ASDR rankings, Zambia ranked first, with an ASMR of 5.17 (95% CI: 3.30–9.53) and an ASDR of 123.81 (95% CI: 74.17–249) (Supplementary Tables 3, 4). Zimbabwe and Cambodia followed, ranking second and third, respectively (Figures 3A,B). Regarding the growth rate of ASMR, the United Arab Emirates (UAE), Lesotho, and Zimbabwe exhibited the most rapid increases, with EAPCs of 2.98 (95% CI: 2.20–3.75), 2.74 (95% CI: 2.29–3.18), and 1.29 (95% CI: 0.84–1.74), respectively (Supplementary Table 5 and Figure 3C). In terms of the growth rate of ASDR, Lesotho ranked first, followed by the UAE and Zimbabwe, with EAPCs of 2.97 (95% CI: 2.48–3.46), 2.16 (95% CI: 1.51–2.81), and 1.59 (95% CI: 1.06–2.12), respectively (Supplementary Table 6 and Figure 3D). Meanwhile, the ASMR and ASDR in the Maldives experienced the most significant decline, with EAPCs of −4.82 (95% CI: −5.10–−4.53) and −5.54 (95% CI: −5.88–−5.19), respectively (Supplementary Tables 5, 6).

### 3.4 DLCACRC burden by age group and sex

**Figure 4:**
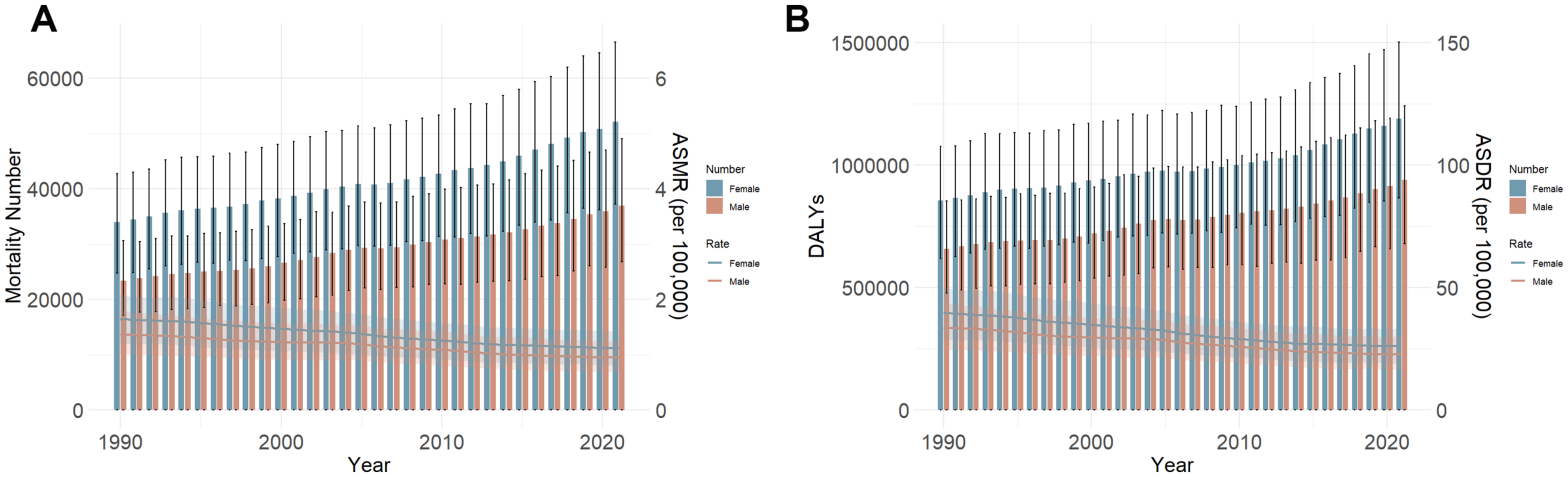
Number and rate of deaths **(A)** and DALYs **(B)** of DLCACRC by sex from 1990 to 2021.

Gender disparities significantly influences the global burden of DLCACRC, disproportionately affecting women. In terms of the number of deaths, DALYs, ASMR, and ASDR, women consistently exhibited higher rates than men (Figures 4A,B). However, from 1990 to 2021, both ASMR and ASDR showed a declining trend in men and women, with no substantial differences in their respective rates of change. The EAPCs for ASMR were −1.26 (95% CI: −1.32–−1.20) for men and −1.37 (95% CI: −1.42–−1.32) for women, while the EAPCs for ASDR were −1.36 (95% CI: −1.41–−1.31) for men and −1.51 (95% CI: −1.57–−1.45) for women (Table 1). In contrast, the number of deaths and DALYs has increased annually, demonstrating an opposite trend to the decline observed in ASMR and ASDR. Moreover, no statistically significant difference in the rates of change was observed between men and women (Figures 4A,B and Table 1).

**Figure 5:**
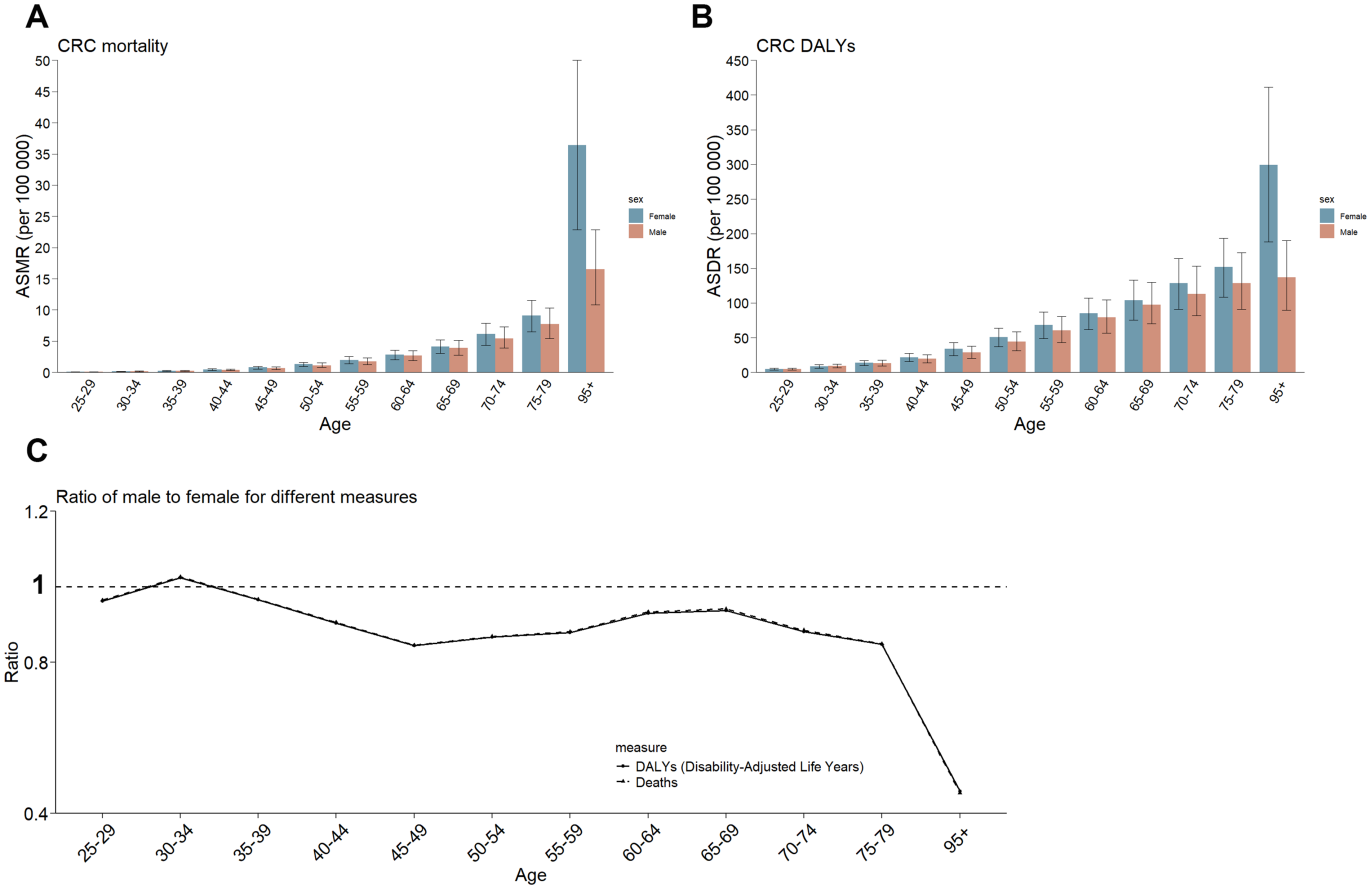
The rate of deaths and DALYs of DLCACRC and its ratio of male to female by age group in 2021. **(A)** The rate of deaths of DLCACRC by age group in 2021. **(B)** The rate of DALYs of DLCACRC by age group in 2021. **(C)** Ratio of male to female of the rate of deaths and DALYs of DLCACRC by age group in 2021.

Data from 2021 indicate that the disease burden of DLCACRC increases with age, with the highest ASMR and ASDR observed in the 95 + age group. In all age groups, both ASMR and ASDR were consistently higher in women than in men (Figures 5A,B). The trends in the male-to-female ratio for both ASMR and ASDR were similar, with the curves nearly overlapping. As age increases, this ratio remains relatively stable, except in the 95+ age group, where the female ratio is significantly higher than the male ratio (Figure 5C).

### 3.5 Correlation between SDI and DLCACRC burden

**Figure 6:**
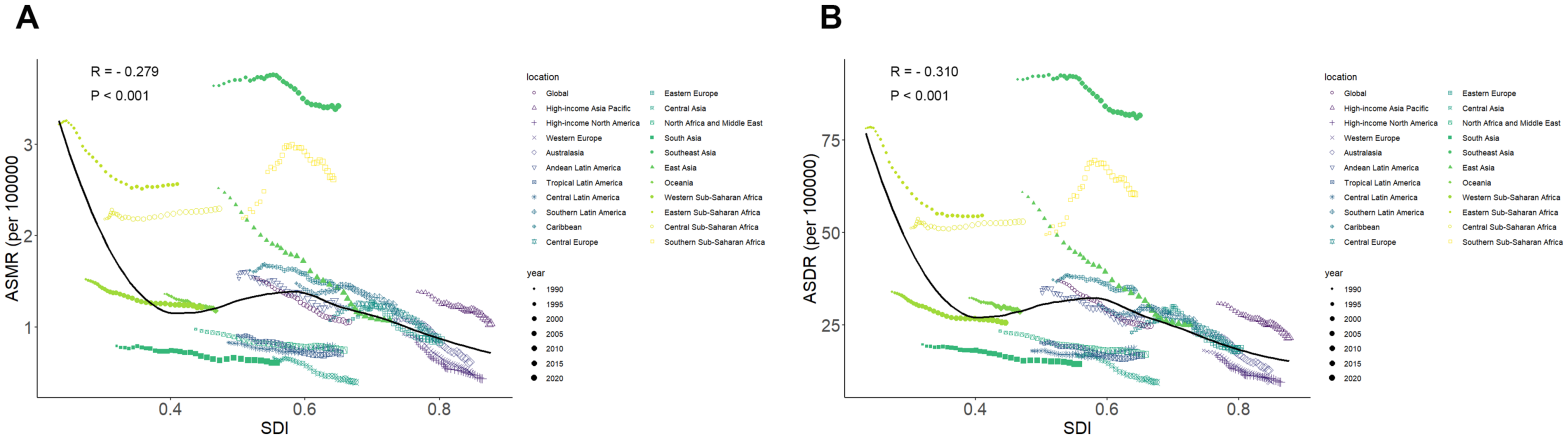
The relationship between ASMR **(A)**/ASDR **(B)** and SDI in 21 regions from 1990–2021.

Data from 2021 showed that the ASMR and ASDR were lowest in high SDI regions, at 0.66 (95% CI: 0.45–0.87) and 13.44 (95% CI: 9.33–17.88), respectively. Conversely, the highest ASMR and ASDR were observed in low SDI regions, at 1.59 (95% CI: 1.18–1.97) and 35.54 (95% CI: 26.48–43.95), respectively (Table 1). However, the relationship between SDI and ASMR/ASDR does not follow a simple linear inverse pattern. Over the past three decades, ASMR and ASDR have demonstrated a non-linear, S-shaped association with SDI. When SDI is below 0.4 or above 0.6, both ASMR and ASDR decline with increasing SDI. However, when SDI falls between 0.4 and 0.6, ASMR and ASDR are positively correlated with SDI (Figures 6A,B).

**Figure 7:**
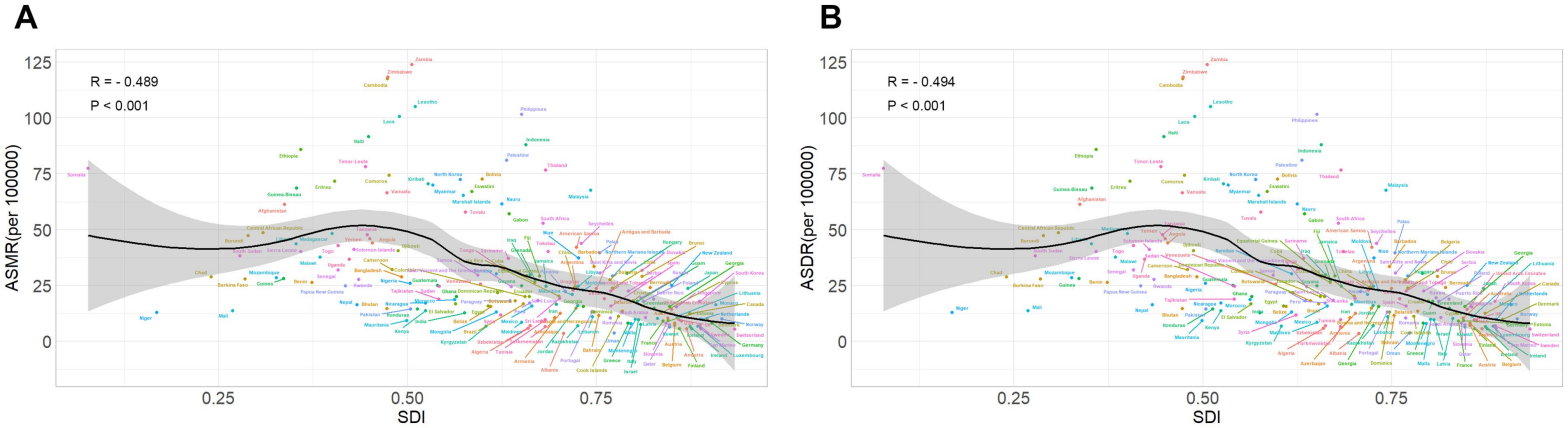
The relationship between ASMR **(A)**/ASDR **(B)** and SDI in 204 countries/territories in 2021.

In 2021, across 204 countries, the relationship between ASMR and SDI for DLCACRC initially exhibited a positive correlation, which gradually decreased as SDI increased. The ASMR reached its highest value when the SDI was approximately 0.42 (Figure 7A). A similar relationship between ASDR and SDI was observed across the same 204 countries (Figure 7B).

## 4 Discussion

Survey data from the GBD database indicated that unhealthy diets had become one of the most significant risk factors for CRC, following behavioral factors, and exert a profound impact on patient prognosis (25). A 2023 study demonstrated that the global burden of CRC attributable to dietary risks has declined over the past three decades (26). Based on the recommendations of the World Health Organization (WHO) and the Food and Agriculture Organization (FAO), the daily dietary calcium requirement is 300–600 mg for infants and children younger than 7 years, and 1,000–1,300 mg for individuals aged 7 years and above (27). Low dietary calcium intake was a common and widespread form of unhealthy diet globally. Numerous studies had confirmed that increased calcium intake can reduce the risk of CRC (14, 28–31). However, the specific mechanisms through which calcium intake influences CRC risk remain unclear; they may involve the abundant expression of calcium-sensing receptors (CaSRs) in normal colonic epithelial cells (14). Calcium was thought to promote the differentiation of colorectal epithelial cells and restore normal colorectal crypt architecture in patients with sporadic adenomas, thereby underscoring the preventive role of calcium intake in CRC (32). This study systematically examined global prevalence trends of DLCACRC and, through stratified and correlation analyses, provided new insights into the disease burden associated with this modifiable dietary risk factor.

From the perspective of global trends, the number of deaths and DALYs from DLCACRC has increased steadily over time, with a cumulative rise of approximately 50%. In contrast, ASMR and ASDR had shown a declining trend. This increase in absolute numbers may be related to the continued growth of the global population over the past three decades (33). The decline in ASMR and ASDR may indicate that the global disease burden of DLCACRC had been reduced. Joinpoint regression analysis indicated that ASMR and ASDR declined most rapidly between 2004 and 2007, followed by a gradual stabilization. According to the 2024 report by the 57th Session of the United Nations Commission on Population and Development, global urbanization accelerated from 1950 to 2007, and subsequently began to slow (34). Notably, between 2004 and 2007, the rapid decline in ASMR and ASDR associated with low-calcium intake may be partially attributed to changes in dietary patterns driven by urbanization. Specifically, oxalic acid, phytic acid, and phosphoric acid in plant foods such as cereals and vegetables will form insoluble salts, affecting calcium absorption; while amino acids released during protein digestion, such as lysine, tryptophan, histidine, arginine, and leucine, can form soluble calcium salts with calcium, thereby promoting calcium absorption (27, 35). With the progression of urbanization, the dietary structure of residents has improved, characterized by decreased cereal consumption and increased intake of meat and dairy products, thereby providing greater protein availability (36, 37). These factors have jointly promoted the absorption of calcium by residents.

Regions with a high SDI are typically more urbanized, and exhibit a relatively lower burden of DLCACRC. In these areas, both the ASMR and ASDR are the lowest among the five SDI regions. However, the determinants of DLCACRC burden extend beyond urbanization alone. From a global perspective, both the ASMR and ASDR for DLCACRC exhibit an “S”-shaped relationship with SDI. Southeast Asia and sub-Saharan Africa exhibit the highest ASMR and ASDR, with sub-Saharan Africa, in particular, experiencing an upward trend in ASMR and ASDR, in contrast to the downward trends observed in most other regions. National-level analyses indicate that Zambia and Zimbabwe report the highest ASMR and ASDR, while Lesotho exhibits the fastest growth rate, all within sub-Saharan Africa. This highlights sub-Saharan Africa as the region with the highest burden of DLCACRC. A large-scale study by Harvard University, analyzing dietary data from over 7 billion individuals across 185 countries, found that inadequate calcium intake was most prevalent in South Asia, sub-Saharan Africa, and East Asia and the Pacific (38). Furthermore, a 2017 systematic review of global calcium intake among adults reported that calcium consumption in Southeast Asia was below 400–500 mg/day, marking the lowest levels worldwide (35). These findings align with the results of the present study, suggesting that low calcium intake in Southeast Asia and sub-Saharan Africa may be attributed to limited dietary diversity, low dairy consumption, and high intake of processed foods (39–42).

From the perspective of gender differences, the number of DLCACRC deaths, DALYs, ASMR, and ASDR all indicated that women are more affected than men, suggesting that the disease burden is significantly greater in women than in men. Reports on sex differences in calcium intake vary across countries. A systematic review of global dietary calcium intake among adults found that women had lower calcium intake than men in 36 of 42 countries reporting sex-specific data (16). This suggested that, globally, women generally consume less calcium than men. Moreover, during specific physiological stages—such as menstruation, pregnancy, lactation, and menopause—women experience increased calcium requirements and greater calcium loss, further affecting their overall calcium balance (43, 44). Despite these observations, there is currently a lack of research exploring whether women with insufficient calcium intake face a higher risk of CRC due to biological or lifestyle-specific mechanisms.

The disease burden of DLCACRC increases with age in both males and females. Both the ASDR and ASMR are significantly higher in older adults compared to younger and middle-aged individuals. Calcium requirements vary across age groups, primarily influenced by differences in bone mass status. Older adults require more calcium due to poorer bone mass status (45, 46). Additionally, the rapid growth of bone mass in young adults during adolescence and the elevated calcium demands during pregnancy and lactation in females further contribute to higher calcium intake requirements (47, 48). Recommendations for calcium intake from various countries generally highlight the highest needs during adolescence and old age. These findings align with the present study, which observes a progressive increase in the DLCACRC burden among older individuals (39). The lower disease burden observed in adolescence may be attributed to the lower incidence of CRC and higher survival rates in this age group.

In summary, Southeast Asia and sub-Saharan Africa bear a significant high burden of DLCACRC, with average calcium intake well below the internationally recommended levels. To enhance calcium intake, it was essential to improve dietary patterns. Countries in Southeast Asia and sub-Saharan Africa should strengthen public health education, promote nutrition-focused initiatives, encourage the consumption of calcium-rich foods—such as dairy products, soy-based foods, and seafood—and improve overall dietary diversity. For low-income countries, where improving dietary structure is both difficult and costly, calcium supplementation with tablets offers a simple and economical solution. Additionally, in countries with a relatively lower overall disease burden, the development of calcium supplementation policies tailored to women should be prioritized, given their disproportionately higher burden of DLCACRC. Particular attention should be paid to calcium intake during physiologically critical periods in women, with timely dietary adjustments aimed at reducing their disease burden. Meanwhile, the significantly higher disease burden observed among the elderly ought not to be overlooked. Targeted calcium supplementation strategies for older adults should be considered a public health priority across all countries. Given the substantial challenges elderly individuals face in modifying dietary habits, the promotion of easily absorbable calcium supplements may represent a more practical and effective strategy.

However, this study has several limitations. First, regarding the GBD database, variations in data collection methods, coding systems, and source quality have resulted in inconsistent data availability across countries and regions. In some regions, poor data quality may introduce a cascading bias effect on model-generated data. Specifically, several less-developed countries lack comprehensive dietary assessments and cancer registries, resulting in data that are often extrapolated from neighboring regions. This study primarily focuses on less-developed regions, the potential for systematic bias is elevated. Second, with the ongoing development of society, dietary structures are becoming increasingly complex, making it more difficult to quantify nutrients accurately, which may reduce the reliability of survey data. Additionally, there remains a lack of authoritative theoretical explanations for the association between low-calcium diets and CRC. The lack of information on other behavioral factors potentially influencing diet and lifestyle may have introduced residual confounding, thus compromising the reliability of the study outcomes. As a longitudinal observational study, this research can only infer associations, rather than establish causal relationships. Collaborative analyses incorporating data from additional databases may represent a viable approach to mitigate this limitation. Finally, as a univariate analysis of CRC, this study only evaluates the association between low-calcium intake and CRC outcomes. Potential confounding factors—such as other dietary components or behavioral habits commonly observed among individuals with low calcium intake—have not been fully addressed, which may limit the interpretability of the findings.

## 5 Conclusion

From 1990 to 2021, the global disease burden of DLCACRC declined, although substantial regional disparities persist. The highest disease burden was concentrated in regions with low levels of social development, particularly Southeast Asia and southern sub-Saharan Africa. Notably, Zimbabwe appears to bear the highest national burden of DLCACRC globally. Countries in these regions should implement targeted public health interventions to increase dietary calcium intake, with feasible strategies including the promotion of dairy consumption. Particular attention should be given to women and older adults, whose physiological characteristics necessitate prioritized nutritional monitoring and timely dietary adjustments.

## Data Availability

The original contributions presented in the study are included in the article/supplementary material, further inquiries can be directed to the corresponding authors.
